# The effects of adolescent methamphetamine exposure

**DOI:** 10.3389/fnins.2015.00151

**Published:** 2015-04-29

**Authors:** Jordan M. Buck, Jessica A. Siegel

**Affiliations:** Department of Psychology, Sewanee: The University of the SouthSewanee, TN, USA

**Keywords:** methamphetamine, adolescence, behavior, cognition, dopamine

## Abstract

Methamphetamine use among adolescents is a significant social and public health concern. Despite increased awareness of methamphetamine use among younger people, relatively little research has examined the effects of adolescent methamphetamine use compared to adult use. Thus, much remains to be learned about how methamphetamine alters adolescent brain function and behavior. In this article we review recent trends in adolescent methamphetamine use and data examining the effects of adolescent methamphetamine use on the dopaminergic system and behavior in humans and animal models. Future research is warranted to expand our understanding of the effects of adolescent methamphetamine exposure and how those effects differ from those seen in adults.

## Introduction

Methamphetamine (MA) use and abuse is a major public health concern in the United States (NIDA, 2006, 2013; DASIS, 2008). MA's primary mechanism of action is on the brain dopamine (DA) system, resulting in high abuse potential, neurotoxic effects on the DA system, and behavioral and cognitive impairments in adults (Sulzer et al., 2005). Historically, MA was primarily used by adults and was not commonly used by adolescents, but in more recent years the demographic profile of MA use has widened to include adolescents (Rawson et al., 2007; Gonzales et al., 2010).

Epidemiological research suggests that compared to other drugs of abuse, adolescent MA use is relatively low in the United States. The most contemporary data from the Monitoring the Future Survey in 2013 shows past-year MA use among 8th and 10th graders at 1.0% and among 12th graders at 0.9% (Johnston et al., 2013). These current rates of MA use among youth are lower than the initial measurements obtained in 1999, suggesting overall decreases in adolescent MA use (Johnston et al., 2013). Caution should be taken, however, when interpreting these national trends. While indeed encouraging, other data suggest a more concerning situation. For example, MA-related emergency department visits rose from 67,954 in 2007 to 102,961 in 2011 (DAWN, 2014), suggesting negative and severe consequences of MA among those using the drug. The percentage of adolescents admitted to Los Angeles County adolescent drug treatment centers with MA as their primary drug of abuse has increased since 2002 (8% in 2002 and 31% in 2005) (Gonzales et al., 2008). Adolescent MA users show poorer treatment response and are less likely to remain drug-free during treatment compared to non-MA using adolescents (Rawson et al., 2005). Thus, while downward national trends in self-reported adolescent MA use are promising, adolescent MA use remains an issue that warrants attention.

While much research has focused on the effects of adult MA use, relatively little research has examined the effects of MA use in adolescents. As adolescence is a dynamic period of brain and behavior development (Spear, 2000), it is important that we better understand the effects of MA exposure on the adolescent brain and how these effects may differ from those seen in adults. This review will summarize the literature examining the effects of adolescent MA use in humans and animal models and briefly compare these results to those found in adults.

## The effects of adolescent MA use

MA use during adolescence causes distinct effects from other drugs of abuse. Adolescents in treatment for MA abuse have an increased likelihood of a history of psychiatric treatment and a family history of drug misuse (Miura et al., 2006). Similarly, Rawson et al. (2005) found that adolescents in treatment for MA abuse show increased rates of depression and suicide ideation compared to adolescents in treatment for other drugs of abuse (Rawson et al., 2005). Adolescent MA users are more likely to have had previous treatments for drug abuse compared to adolescents using other drugs of abuse (Gonzales et al., 2008). Adolescent MA users are also more likely to be female, which differs from most other abused drugs (Rawson et al., 2005; Miura et al., 2006; Gonzales et al., 2008). MA use during adolescence is associated with increased rates of risky sexual behavior and adolescent pregnancy, as well as behavioral problems such as increased anti-social behaviors (Zapata et al., 2008; Embry et al., 2009).

The MA-induced psychological and behavioral alterations in adolescent users appear to persist even after the termination of MA use, as abstinent adolescent MA users show increased depression and anxiety scores and increased cortisol secretion following a social stressor compared to non-MA users after 4–11 months of abstinence (King et al., 2010b). Abstinent adolescent MA users also show executive function impairments after 4–11 months of abstinence compared to non-MA using adolescents (King et al., 2010a). The long-term effects of MA in adolescent users parallel certain long-term effects observed in abstinent adult MA users, who also show impairments in executive functions (for a review, see Scott et al., 2007), increased levels of depression, and increased anxiety (Zweben et al., 2004; Salo et al., 2011; Li et al., 2013). However, the number of studies on adolescent MA users is limited compared to those in adults, and the time of abstinence in the adolescent studies is relatively short (4–11 months), necessitating further research to understand the potential severity and duration of the long-term effects of adolescent MA use.

To the best of our knowledge, very few studies have assessed the effects of MA on functional or anatomical brain measures in adolescent users. Sung et al. (2013) examined brain levels of N-acetylaspartate plus N-acetylaspartyl glutamate (tNAA) and phosphocreatine plus creatine (PCr+Cr) ratios, which are brain metabolite markers of neuronal viability and integrity, in current adolescent MA users. The authors found that MA users do not show significant changes in mid-frontal gray matter tNAA/ PCr+Cr ratios (Sung et al., 2013). This differs from abstinent adult MA users, who show significantly lower brain tNAA levels (Nordahl et al., 2002). However, there is a significant negative correlation between tNAA/PCr+Cr and the duration of MA use in adolescent MA users (Sung et al., 2013). Lifetime doses of MA in adolescent users are also positively correlated with the size of the left putamen and novelty seeking behavior (Churchwell et al., 2012). Taken together, these data suggest that certain MA-induced brain changes in adolescent users may be similar to those found in adults, but much more research assessing other aspects of brain function in adolescent MA users is required in order to delineate how MA alters the adolescent brain.

## Effects of adolescent MA use in animal models

Few studies have assessed functional and structural brain measures in adolescent MA users. Thus, preclinical research has been crucial for our understanding of the effects of MA exposure on the adolescent brain and how these effects may differ from those in the adult brain. While it appears that some of the cognitive, behavioral, and psychological effects of MA are similar between adolescent and adult users, animal studies suggest that the adolescent brain is partially protected against the neurotoxic effect of MA on the DA system compared to the adult brain. For example, adolescent male mice at 1 month of age exposed to a high dose of MA (4 × 10 mg/kg) show 50% reductions in striatal DA levels 72 h post exposure, whereas adult mice at 12 months of age exposed to the same dose of MA show 80% reductions in striatal DA levels (Miller et al., 2000). Adolescent rats exposed to high doses of MA (4 × 10 mg/kg) on postnatal day (PND) 40 show no changes in DA uptake, dopamine transporter (DAT) binding, DAT activity, and tyrosine hydroxylase activity in the striatum 7 days after exposure, whereas these dopaminergic measures are reduced following the same doses of MA in adult rats exposed on PND 90 (Kokoshka et al., 2000; Riddle et al., 2002). Biweekly injections of MA (7.5 mg/kg) for 6 weeks starting in adolescence on PND 40 attenuates MA-induced decreases in striatal vesicular DA uptake in adulthood on PND 90 in rats (McFadden et al., 2011). High doses of MA (4 × 10 mg/kg) in adolescent rats on PND 40 moderately reduce vesicular DA uptake 1 h post exposure, whereas the effects of MA on vesicular DA uptake in the adult rat on PND 90 are significantly more severe 1 h post exposure (Truong et al., 2005). This dose of MA also does not reduce striatal DA levels or vesicular DA uptake 1 week after exposure in adolescent rats, whereas these measures are reduced 1 week post-treatment in adult rats (Truong et al., 2005). In contrast, Kokoshka et al. (2000) showed that reductions in striatal DA uptake and DAT activity occur in both adolescent and adult rats 1 h post MA exposure, suggesting that the immediate effects of MA are similar amongst adolescents and adults, but that these effects mitigate more rapidly in the adolescent brain (Kokoshka et al., 2000). Interestingly, Kokoshka et al. (2000) also showed that the effect of MA on the serotonin system, as measured by reductions in tryptophan hydroxylase activity, are present in both adolescent and adult rats at 1 h and 7 days post MA exposure (Kokoshka et al., 2000). Similarly, MA exposure increases cFos activity in a variety of brain regions to comparable levels in both adolescent and adult mice (Zombeck et al., 2010). These findings suggest that adolescent and adult MA exposure causes indiscriminate impairments in serotonergic function and activation of various brain regions, in contrast to the apparent age-specific effects exerted by MA on the DA system.

The mechanisms underlying the relative resistance to the effects of MA on the DA system in the adolescent rodent brain are not well understood. Importantly, the attenuated toxicity of MA in adolescents vs. adults is not due to differences in MA-induced hyperthermia, as both adolescent and adult rats show comparable increases in body temperature following MA exposure (Kokoshka et al., 2000; Truong et al., 2005). The relative resistance in adolescence to the neurotoxic effects of MA on the DA system may be due in part to developmental differences in the DA system. Adolescent rodents show increased levels of functionally active DAT and increased vesicular monoamine transport of DA into vesicles compared to adult rats (Volz et al., 2009). The DA D1 and D2 receptors show increased expression in the striatum and nucleus accumbens until PND 40 in adolescence, when they begin to decrease as adulthood is reached, which may reflect the pruning of excess dopaminergic synapses during this developmental time period (Teicher et al., 1995). Alternatively, pharmacokinetic factors may mediate the age-dependent effects of MA in the rodent brain. Plasma and striatal levels of MA are much higher in adult rats on PND 90 compared to adolescent rats on PND 40 1 h after MA exposure (Kokoshka et al., 2000). In contrast, Zombeck et al. (2009) found little to no difference in brain MA concentrations between adolescent and adult mice up to 240 min post MA exposure, suggesting similar peak levels of MA and a similar pharmacokinetic time course in both age groups (Zombeck et al., 2009). These conflicting data demonstrate that more research is needed to determine if pharmacokinetic differences mediate the differential effects of MA on the DA system in adolescent vs. adult rodents.

The effects of MA on the hypothalamic pituitary-adrenal (HPA) axis are of particular interest, as adolescent MA users show increased rates of depression, suicide ideation, anxiety, and enhanced cortisol release following a stressor compared to controls (Rawson et al., 2005; King et al., 2010b). As MA-induced alterations in the HPA axis and stress responsiveness could have significant effects on other brain neurotransmitter systems and behavior, it is important to model the potential MA-induced changes in the HPA axis in adolescent animals. MA exposure during early adolescence (4 × 7.5 mg/kg on PND 30 and 31) decreases vasopressin-immunoreactive neurons in the paraventricular nucleus of the hypothalamus later in adolescence in male mice (Joca et al., 2014). Unpublished findings from our lab also suggest that early adolescent MA exposure tends to reduce serum corticosterone levels later in adolescence. Although these findings did not reach statistical significance, further research is required to characterize the effects of adolescent MA exposure on HPA axis function.

Much research has examined the behavioral and cognitive effects of MA in adult rodents. However, relatively few studies have examined the behavioral and cognitive effects of MA exposure in adolescent rodents. Joca et al. (2014) modeled the increased rates of depression among human adolescent MA users, showing that early adolescent MA exposure (4 × 7.5 mg/kg on PND 30 and 31) increases depression-like behavior in the Porsolt forced swim test in late adolescent male and female mice, an effect that persisted into adulthood (Joca et al., 2014). However, the authors did not examine the effects of adult MA exposure in this study, rendering it difficult to compare this behavioral effect of MA to an adult age group. Nonetheless, these findings model the increases in depression rates and suicide ideation found in adolescent human MA users (Rawson et al., 2005). In order to model and better understand the effects of human adolescent MA use, additional research is warranted to assess the effects of adolescent MA exposure on depression and anxiety behaviors and related brain circuits.

Other research examining the behavioral effects of MA exposure in adolescent and adult rodents has shown that MA can exert unique effects in adolescence. Adolescent mice (PND 30-35) show more moderate increases in locomotor activity up to 60 min (Zombeck et al., 2009) or up to 90 min (Zombeck et al., 2010) following an injection of MA (2 mg/kg) compared to adult mice (PND 69-74). Furthermore, 4 mg/kg of MA results in prolonged increases in locomotor activity for 100 min post injection in adult mice (PND 69-74), whereas the increased locomotor activity in adolescent mice (PND 30-35) begins to decline approximately 60 min post-treatment (Zombeck et al., 2010). Similarly, repeated exposure to 0.5 mg/kg MA increases locomotor activity in both adolescent and adult rats, but these increases are more moderate in the adolescent rats (PND 34-38) compared to the adults (PND 66-70) over 5 days of exposure (Zakharova et al., 2009). In contrast to the mitigated effects of MA on locomotor activity in adolescent vs. adult rodents, adolescent rats are more susceptible to the conditioned rewarding effects of MA than adults. Adolescent rats express MA-induced conditioned place preference (CPP) following just 3 days of training, while adult rats require 5 days of training to develop MA-induced CPP (Zakharova et al., 2009).

Minimal research has examined the effects of adolescent MA exposure on cognitive function in animal models. Exposure to MA from PND 41-50 during adolescence (6.5 mg/kg/day) results in spatial learning impairments in the Morris water maze and sequential learning impairments in the Cincinnati water maze. However, exposure to MA at younger ages prior to adolescence (PND 21-30 and PND 31-40) and older ages in adulthood (PND 51-60) does not impair performance on these cognitive tests, suggesting enhanced vulnerability during adolescence to the deleterious cognitive effects of MA (Vorhees et al., 2005). Adolescent MA exposure (10 ml/kg from PND 41-50) results in attenuated visual discrimination and impaired reversal learning later in adulthood in rats, and also increases the amount of MA that adult rats will self-administer (Ye et al., 2014). Exposure to MA during early adolescence (4 × 7.5 mg/kg on PND 30 and 31) in mice, however, does not impair cognitive function in the Morris water maze or the novel object recognition test later in adolescence or in adulthood (Joca et al., 2014). Adolescent mice also lack short-term memory impairments and alterations in hippocampal plasticity following neurotoxic doses of MA (24 mg/kg/day for 14 days), but this MA exposure paradigm does impair spatial memory and hippocampal plasticity after 21 days of abstinence from the drug, suggesting that MA-induced impairments may only appear after a period of abstinence in adolescence (North et al., 2013). Conflicting data assessing the cognitive effects of adolescent MA exposure in animal models necessitate further exploration of this topic. However, the MA-induced cognitive impairments found by Vorhees et al. (2005) and Ye et al. (2014) indicate that at the preclinical level MA may lead to long-term impairments in a variety of cognitive domains. These findings should be further explored to assess the potential for similar impairments in humans following adolescent MA use.

When considering the limited number of studies on adolescent MA exposure in animal models, it is interesting to note that despite the relative resistance of the adolescent DA system to the neurotoxic effects of MA, adolescent rodents nevertheless show MA-induced behavioral and cognitive changes and impairments that are concerning from a translational perspective. It is unclear what MA-induced brain changes may underlie the MA-induced behavioral and cognitive alterations in adolescence. While Joca et al. (2014) and Kokoshka et al. (2000) showed MA-induced impairments in the vasopressin system in the paraventricular nucleus of the hypothalamus and the tryptophan hydroxylase system, respectively, further research is required to understand MA-induced brain changes in adolescence and how these changes result in the behavioral and cognitive impairments noted in the studies above. It is also important to note the broad spectrum of dosages and exposure paradigms utilized in studies examining the effects of adolescent MA confound efforts to conclusively understand the effects of adolescent MA exposure. Studies use chronic or acute MA exposure paradigms, a variety of MA doses, as well as diverse MA administration methods (inter-peritoneal, intravenous, intra-cerebral injection, etc.). Furthermore, distinctions must be made between self-administration models and experimenter-administered models, as the behavioral, cognitive, and neurochemical outcomes of each may differ in adolescents. The broad range of experimental designs in MA exposure studies makes it challenging to find consistent results, and future research should replicate previous exposure paradigms and emphasize more standardized exposure models to enable more meaningful comparisons among studies examining the effects of adolescent MA exposure.

## Conclusion

In summary, the current literature suggests that adolescent MA use results in increased risky sexual behaviors and psychiatric problems in humans, some of which are modeled in preclinical research. Animal studies indicate that adolescent MA exposure impairs cognitive function and can increase MA use later in life, giving cause for concern in terms of what we might expect to see in human MA users who initiated use during adolescence. However, the brain basis of these impairments is yet to be determined. Interestingly, the brain DA system seems to be relatively resistant to the effects of MA during adolescence as compared to during adulthood. This may arise due to developmental changes in the DA system during adolescence, and further research is warranted to better understand these mechanisms. Research examining the effects of adolescent MA exospore in animal models is limited in scope compared to the research examining the effects of adult MA exposure. This necessitates further research that delineates the potentially unique brain and behavioral changes elicited by MA exposure in adolescence.

## Author contributions

JB and JS were both involved in the writing and conceptualization of the manuscript. Both authors contributed to the editing and approval of the final version.

### Conflict of interest statement

The authors declare that the research was conducted in the absence of any commercial or financial relationships that could be construed as a potential conflict of interest.
